# Pacemaker with automatic activation of a magnetic resonance imaging mode: A single-center experience

**DOI:** 10.1016/j.ahjo.2026.100732

**Published:** 2026-01-28

**Authors:** Bridget McIlraith, Justin A. Mariani, Ross Downey, Geoffrey Clare, Matthew Daly, Houda El Banna, Ian Crozier

**Affiliations:** a Department of Cardiology, Christchurch Hospital, Christchurch, New Zealand; b Department of Cardiology, Alfred Hospital, Australia; c Heart Failure Research Group, Baker Heart and Diabetes Institute, Department of Medicine, Monash University, Australia

**Keywords:** MRI-conditional pacemaker, Cardiac implantable electronic device, Amvia, MRI Guard 24/7, Workflow efficiency, Automatic mode switching

## Abstract

**Introduction:**

In patients with permanent pacemakers, magnetic resonance imaging (MRI) traditionally requires resource-intensive manual device reprogramming before and after the scan putting demands on clinical resources.

**Methods:**

In this single-center case series, we report five MRI procedures in three patients with a pacemaker using an always-on MRI workflow facilitating feature (MRI Guard 24/7) that automatically detects MRI conditions and adjusts the pacing mode without the need for pre- or post-scan checks.

**Results:**

All scans were completed successfully, with automatic switching into MRI mode and reverting to the permanent setting. No device reconfigurations were required pre- or post-scan.

**Discussion:**

We discuss the potential of this feature to streamline MRI workflows, reduce staffing demands and improve access to timely imaging for patients with cardiac implantable electronic devices.

## 1 Introduction

Magnetic Resonance Imaging (MRI) is a widely used imaging tool in the medical field, owing to its excellent identification of soft tissue without exposure to ionizing radiation. In patients with cardiac implantable electronic devices (CIED), MRI was previously contraindicated due to the risks of device damage and inappropriate triggering or inhibition of stimulation from the high intensity magnetic and electrical fields [1]. Estimates predict one in 50 persons over age 75 has a CIED [1], [2]: 75% of these will need an MRI during their lifetime, and additionally approximately 17% of patients need multiple scans [1], [2], [3].

Over the last decade, CIED manufacturers have shown that specially designed devices can safely undergo MRI procedures, and “MR conditional” labelling has become a standard of care. While this has significantly reduced safety concerns, the need for temporary programming of the device to asynchronous pacing and deactivation of tachycardia therapy in implantable cardioverter defibrillators during the scan procedure remains a significant barrier to MRI scans in this population, particularly due to the challenges of multi-disciplinary coordination between radiology and cardiology departments. MRI scans in the CIED population have thus increased workload burdens and expenses [3]. Appropriate budgeting, staff allocation, scanning logistics in the medical settings, and standardizing automated CIED pre-programming features among manufacturers have been well documented as strategies to mitigate the hurdles for safe and timely MRI scans for CIED patients [3], [4], [5].

As part of the BIO|CONCEPT Amvia study (NCT05610176), we report on our initial single-center experience with the Amvia pacemakers (Biotronik, Germany), a device family that includes MRI Guard 24/7, a novel feature designed to automatically detect MRI conditions and adjust pacing modalities without requiring pre- or post-scan programming.

## 2 Methods

The BIO|CONCEPT Amvia study is a prospective, multicenter, first-in-human study conducted in Australia and New Zealand. It was designed to evaluate preliminary safety and product performance of the new Amvia pacemaker family (Biotronik, Germany) in patients with bradycardia or cardiac resynchronization therapy pacemaker (CRT-P) indications. Patients who were implanted with an Amvia system were followed for one year, undergoing hospital discharge, 1-month, 3-month, and 12-month post implant follow-up. Remote monitoring was activated for all patients.

**Fig. 1:**
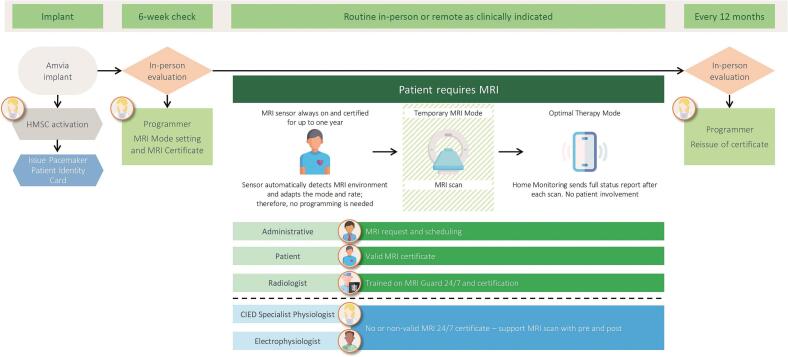
Schematic workflow for MRI scans in patients with pacemakers using the automatic MRI mode switching feature MRI Guard 24/7. After initial system check, MR conditional parameters can be set and maintained for up to one year. The always-on sensor continuously monitors for MRI exposure and automatically switches the device to temporary MRI mode during the scan. Upon exiting the MRI environment, the device automatically reverts to the permanently set pacing mode, and a post-MRI scan reports is transmitted via remote monitoring.

The Amvia pacemakers feature “MRI Guard 24/7”, an algorithm which enhances Biotronik's previously available MRI AutoDetect algorithm. While MRI AutoDetect allowed pre-programming for MRI scans within a 14-day window, MRI Guard 24/7 enables continuous MRI readiness for up to one year following the initial system check (Fig. 1). The system automatically detects MRI environments at a threshold of approximately 140 millitesla (mT) and switches the device to an MRI mode, if the gradient field strength is gradually increasing within 4 s. This temporary mode is the asynchronous equivalent of the permanent setting (e.g., DDD becomes DOO), with the MRI mode either automatically selected by default or manually programmed, to avoid inappropriate stimulation triggered by MRI induced artefact sensing. The pacing rate is calculated from the actual rate before scanner detection plus 15 beats per minute (bpm) to avoid competing intrinsic and stimulated rhythms. On exiting the MRI gradient field at 140 mT the device automatically reverts to the permanent program. This feature allows for MRI scans at any time without the need for dedicated on-site follow-ups to adjust and check the device functionality.

At our tertiary center, 16 patients were implanted with Amvia pacemakers between March and May 2023. Device settings were reviewed, and MRI Guard 24/7 was activated at six weeks post-implantation. Patients referred for MRI at our institution are first triaged by CIED specialist physiologist who determines the MRI conditional status, MRI technologists then protocolize scanning conditions based on manufacturer-specific MRI technical manuals. The communication between cardiology and radiology is via email or phone and documented in patient medical records. Patients who underwent MRI examination were monitored by a CIED specialist physiologist and a radiology nurse. Monitoring during the scan included pulse oximetry and an audiovisual system for continuous observation and communication between scan sequences. Pre- and post-scan device interrogation was performed comprising a full check of lead parameters, underlying rhythm, battery status, and to assess for any adverse effects. The above was performed as per our local standard protocol.

## 3 Results

Between September 2023 and March 2024, three patients implanted with Amvia pacemakers at our center underwent a total of five 1.5 Tesla MRI scans. MRI scans were performed between 3- and 12-month follow-up. All patients were women aged 67–84 years; two had received a dual chamber pacemaker for chronic heart block and one had a CRT-P device for dilated cardiomyopathy. Of the five MRI procedures, four were brain scans and one was a scan of the lumbar spine. The baseline and MRI programs are shown in Table 1. Pulse oximetry confirmed the increased heart rate prior to the scan, which was consistent with the predicted rate for automatic MRI mode under the magnetic field. Asynchronous pacing modes were employed for all scans as the algorithm does not allow for pacing mode “OFF”, CRT pacing was also maintained for the CRT patient. Device interrogation following the scan showed appropriate return to the initial permanent program. No inappropriate activation of MRI mode was observed in any patient outside of an MRI environment. Additionally, in one patient who required three MRI scans within two months, no device reconfiguration was needed pre- or post-scan.

**Table 1:** Patients' CIED programming pre, during, and post magnetic resonance imaging.

Patient	Permanent programming pre-MRI rate	MRI Guard 24/7 mode^1^	Automatically set permanent programming post-MRI rate
NZL003–004	DDD 50–140 bpm	DOO 112 bpm	DDD 50–140 bpm
NZL003–005	DDD-CLS 50–150 bpm	DOO 96 bpm	DDD-CLS 50–150 bpm
NZL003–005	DDD-CLS 60–140 bpm	DOO 80 bpm	DDD-CLS 70–140 bpm
NZL003–005	DDD-CLS 70–140 bpm	DOO 110 bpm	DDD-CLS 70–140 bpm
NZL003–008	DDD 50–120 bpm	DOO 87 bpm	DDD 50–120 bpm

1 The rates represent automatically set individual pacing rates, calculated as the mean heart rate prior to the scan plus 15 bpm.

## 4 Discussion

Current solutions for MRI mode activation in CIEDs can be categorized into automatic and manual/semi-automatic approaches. While automatic systems detect the MRI environment and switch without on-site intervention, manual or semi-automatic approaches require pre- and post-MRI scan activation. MRI Guard 24/7 represents an automatic approach that allows for the setting of MR conditional parameters for up to one year. While the initial applications of the feature that we describe here were closely monitored as per our local protocol, it may allow MRI scans without a CIED specialist.

Due to the rising number of clinical situations where MRI scans are valuable for decision-making and the rising number of CIED implants, increasing human resources have been required for this important diagnostic tool with conventional devices. By avoiding the need for reprogramming of CIEDs, MRI Guard 24/7 reduces the on-day coordination between radiology and cardiology departments and frees the capacity of clinical staff for more urgent care. The increased efficiency may ultimately reduce the waiting time for MRI scans and minimize the length of hospital stays for patients awaiting MRI scans, freeing up hospital beds and reducing overall healthcare costs. MRI Guard 24/7 should facilitate emergency MRI in CIED patients. Finally, the feature avoids the risk of failed or incorrect reprogramming of devices following the MRI scan.

While our initial observations of this feature are promising, the sample size is limited, and all data are from a single center. The workflow improvements are currently applicable only to Biotronik devices with MRI Guard 24/7 enabled and do not extend to ICD-specific considerations such as arrhythmia detection and therapy suspension which require separate evaluation. This limits generalizability, particularly for institutions managing devices from multiple manufacturers. Local protocols and communications must still be followed to ensure patient readiness and safety for MRI. An MRI certificate summarizing device-specific scanning conditions, such as those issued with MRI Guard 24/7-compatible certificates, may support/add value to the referral review by helping confirm device type and MRI conditional status. However, they were not used within our MRI workflow itself, where radiology and cardiology teams rely on device interrogation and manufacturer technical specifications to guide scan eligibility and programming. Further evaluation in larger cohorts and across diverse clinical settings may be necessary to confirm reliability and workflow benefits. Long-term data is another important aspect to assess the device behavior over the full duration of MRI readiness.

In conclusion, automatic switching in MRI mode when being balanced against other logistical and safety considerations has the potential to streamline MRI workflows, minimize opportunities for error, reduce workloads, and enhance patient care. These technologies are welcome additions but must be adopted and integrated safely into clinical real-world practice.

## CRediT authorship contribution statement

**Bridget McIlraith:** Writing – original draft, Validation, Resources, Project administration, Conceptualization. **Justin A. Mariani:** Writing – review & editing, Project administration, Methodology, Investigation, Conceptualization. **Ross Downey:** Writing – review & editing. **Geoffrey Clare:** Writing – review & editing. **Matthew Daly:** Writing – review & editing. **Houda El Banna:** Writing – review & editing. **Ian Crozier:** Writing – review & editing, Supervision, Resources, Project administration, Investigation, Funding acquisition, Conceptualization.

## Ethics statement

This manuscript reports original clinical research conducted in adherence to the Declaration of Helsinki and in compliance with internationally accepted standards for ethical publishing.

## Funding

The study was supported by BIOTRONIK SE & Co. KG, Berlin, Germany.

## Declaration of competing interest

The authors have no conflict of interest to declare.
